# Quantification of Mesenchymal Stem Cells (MSCs) at Sites of Human Prostate Cancer

**DOI:** 10.18632/oncotarget.805

**Published:** 2012-01-13

**Authors:** W. Nathaniel Brennen, Shuangling Chen, Samuel R. Denmeade, John T. Isaacs

**Affiliations:** ^1^ Chemical Therapeutics Program, The Sidney Kimmel Comprehensive Cancer Center at Johns Hopkins, Baltimore, MD, USA

**Keywords:** Mesenchymal Stem Cells, MSC, Inflammation, Prostate Cancer, Drug Delivery, CAF

## Abstract

Circulating bone marrow-derived Mesenchymal Stem Cells (BM-MSCs) have an innate tropism for tumor tissue in response to the inflammatory microenvironment present in malignant lesions. The prostate is bombarded by numerous infectious & inflammatory insults over a lifetime. Chronic inflammation is associated with CXCL12, CCL5, and CCL2, which are highly overexpressed in prostate cancer. Among other cell types, these chemoattractant stimuli recruit BM-MSCs to the tumor. MSCs are minimally defined as plastic-adhering cells characterized by the expression of CD90, CD73, and CD105 in the absence of hematopoietic markers, which can differentiate into osteoblasts, chondrocytes, and adipocytes. MSCs are immunoprivileged and have been implicated in tumorigenesis through multiple mechanisms, including promoting proliferation, angiogenesis, and metastasis, in addition to the generation of an immunosuppressive microenvironment. We have demonstrated that MSCs represent 0.01-1.1% of the total cells present in core biopsies from primary human prostatectomies. Importantly, these analyses were performed on samples prior to expansion in tissue culture. MSCs in these prostatectomy samples are FAP-, CD90-, CD73-, and CD105-positive, and CD14-, CD20-, CD34-, CD45-, and HLA-DR-negative. Additionally, like BM-MSCs, these prostate cancer-derived stromal cells (PrCSCs) were shown to differentiate into osteoblasts, adipocytes, & chondrocytes. In contrast to primary prostate cancer-derived epithelial cells, fluorescently-labeled PrCSCs & BM-MSCs were both shown to home to CWR22RH prostate cancer xenografts following IV injection. These studies demonstrate that not only are MSCs present in sites of prostate cancer where they may contribute to carcinogenesis, but these cells may also potentially be used to deliver cytotoxic or imaging agents for therapeutic and/or diagnostic purposes.

## INTRODUCTION

The prostate is subjected to numerous infectious and inflammatory insults over the course of a man's lifetime, ranging from dietary carcinogens to physical trauma to viral and bacterial pathogens [1]. In fact, greater than 80% of men have evidence of inflammation in their prostate at biopsy [2]. Furthermore, prostatitis likely effects all men at some point during their life, at least acutely [1-2]. While many of these inflammatory lesions will be resolved naturally without intervention, a subset of these will go on to develop clinical symptoms as a result of chronic inflammation. Chronic inflammation has been suggested as an initiating event in prostate carcinogenesis as evidence of a leukocytic infiltrate is frequently present at sites of prostatic intraepithelial neoplasia (PIN) and proliferative inflammatory atrophy (PIA), prostate cancer precursor lesions [1].

Mesenchymal stem cells (MSCs) are adult stem cells that have recently gained attention as potent modulators of both the innate and adaptive immune responses [3-5]. MSCs have been minimally defined by the International Society for Cell Therapy (ISCT) as adult stem cells of fibroblastoid morphology that can adhere to tissue culture plastic, express CD73, CD90, and CD105 in the absence of hematopoietic lineage markers, including CD11b, CD14, CD19, CD34, CD45, CD79a, and HLA-DR [4, 6-7]. Additionally, these cells have the ability to differentiate into cells of the mesoderm lineage, including adipocytes, chondrocytes, and osteoblasts [6], but may also include additional cell types such as pericytes [4, 8-9], myocytes [9-10], and neurons [11-13], though the latter is the subject of controversy [14-15]. Due to the lack of HLA-DR expression and the associated co-stimulatory molecules, MSCs are immunoprivileged and thus escape immune surveillance [3-4, 16]. Furthermore, MSCs have been shown to mediate immunosuppression through multiple mechanisms involving nearly every component of the immune system, both the innate and adaptive arms [3-5]. MSCs traffic to sites of inflammation through the action of soluble chemokines and cytokines emanating from these lesions [17-19]. MSCs have been shown to express a great number of the cognate receptors for these chemokines and cytokines, which have been shown to mediate their homing properties [17].

This latter point is particularly relevant, because the prostate has frequently been shown to contain sites of inflammation, and prostate cancer expresses high levels of pro-inflammatory stimuli, including CXCR4, CCL5, and CCL2 [18, 20-22]. In 2007, Lin et al. characterized stromal cells from benign prostatic hyperplasia (BPH) tissue that had multi-lineage differentiation potential consistent with MSCs [23]. However, because these stromal cells lacked the ability to differentiate into neurons, the authors concluded that these cells did not represent MSCs [23]. In 2010 and 2012, however, it was demonstrated that the ability of MSCs to differentiate into neuronal cells is highly dependent on the age of the donor [13, 24]. These studies documented that MSCs derived from older donors (>45) lose the ability to differentiate into neuronal cells [13, 24]. Therefore, since the Lin et al. study utilized BPH tissue from patients older than 45, this differentiation potential would be consistent with MSCs derived from older donors. In the data presented herein, we demonstrate that a population of cells can be isolated from primary prostate cancer specimens prior to expansion in tissue culture that is consistent with an MSC phenotype. These primary prostate cancer stromal cells or PrCSCs are FAP-, CD90-, CD105-, and CD73-positive in the absence of CD14, CD20, CD34, CD45, and HLA-DR expression. Furthermore, a subset of these cells is able to differentiate into osteoblasts, adipocytes, and chondrocytes; thereby, demonstrating their multipotent nature. Like bone marrow-derived MSCs (BM-MSCs), these PrCSCs can traffic to sites of prostate cancer in vivo.

## RESULTS

### Multi-lineage Differentiation Potential of Human Prostate Cancer-derived Stromal Cells

**Figure 1:**
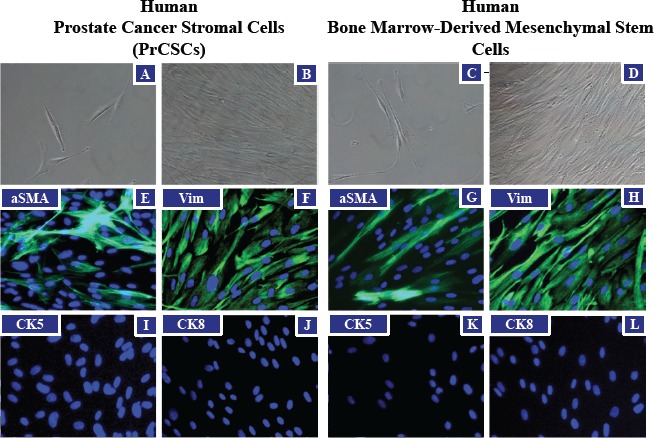
Morphological Similarities between PrCSCs and hBM-MSCs Prostate cancer-derived stromal cells (PrCSCs) and human bone marrow-derived mesenchymal stem cells (hBM-MSCs) have similar morphologies at low (A and C) and high (B and D) densities (representative phase-contrast images). Both PrCSCs and hBM-MSCs stain positive for mesenchymal markers, alpha-smooth muscle actin (aSMA) (green, E and G) and vimentin (Vim) (green, F and H), but not epithelial markers, cytokeratin 5 (I and K) or cytokeratin 8 (J and L) by immunofluorescence. Nuclei counterstained with DAPI (blue, E-L).

**Figure 2:**
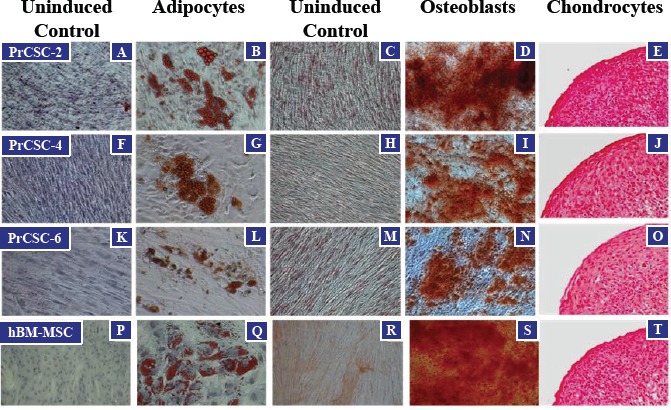
Multi-lineage Differentiation of PrCSCs and hBM-MSCs PrCSCs derived from multiple patients (PrCSC-2, -4, -6) are able to differentiate into adipocytes (B, G, and L), osteoblasts (D, I, and N), and chondrocytes (E, J, and O) when placed in the appropriate induction media as defined by positive staining for lipid vacuoles (adipocytes, Oil Red O), calcium mineralization (osteoblasts, Alizarin Red S), and glycosaminoglycans (chondrocytes, Safranin-O), respectively. Differentiation indicated by red staining in each. In contrast, no differentiation is observed when these cells are not cultured in the presence of the various inducing factors (adipocytes: A, F, K, and P; osteoblasts: C, H, M, and R). Differentiation into these three lineages is one of the defining characteristics of mesenchymal stem cells as demonstrated by the hBM-MSC positive controls (Q, S, and T).

Tissue cores of human prostatectomy specimens were obtained immediately following surgery, dissociated into a single cell suspension, and placed in tissue culture (RPMI) media supplemented with 10% fetal bovine serum (FBS). From these explanted cells, outgrowth of fibroblast-like prostate cancer-derived stromal cells (PrCSCs) (Figure 1A, B) was observed that had a similar morphology to human bone marrow-derived MSCs (hBM-MSCs) (Figure 1C, D). If a portion of the same cellular suspension was cultured in keratinocyte serum-free media (K-SFM), basal-like prostate-derived epithelial cells (PrECs) were obtained [25-28]. Both hBM-MSCs and PrCSCs stained positive for alpha-smooth muscle actin (aSMA) (Figure 1E, G) and vimentin (Vim) (Figure 1F, H), but not cytokeratins 5 (CK5) (Figure 1I, K) or 8 (CK8) (Figure 1J, L). These results are the absolute opposite of those obtained for PrECs, which are negative for aSMA and Vim, but positive for CK5 and CK8 [25-28]. Similar to hBM-MSCs (Figure 2Q, S, T), differentiation of PrCSCs into adipocytes (Oil Red O-positive) (Figure 2B, G, L), osteoblasts (Alizarin Red-positive) (Figure 2D, I, N), and chondrocytes (Safranin O-positive) (Figure 2E, J, O) was observed if the cells were cultured under the appropriate induction conditions, but not in the uninduced controls (Figure 2A, C, F, H, K, M, P, R). Furthermore, these cells were shown to be fibroblast activation protein (FAP)^+^, CD90^+^, CD105^+^, CD73^+^, and alpha-smooth muscle actin (aSMA)^+^ by flow cytometry in the absence of CD45, CD34, CD11b, CD19, and HLA-DR expression (Table 1). In contrast, PrECs do not differentiate into these cell types under the same conditions (data not shown). Importantly, only a subset of cells within PrCSCs derived from a single donor possesses this tri-lineage differentiation potential (Figure 2A-O). In addition, not all PrCSCs derived from different patients were able to differentiate into all lineages (Table 1). Interestingly, the multi-lineage differentiation potential of the PrCSCs does not appear to correlate with Gleason Score (Table 1).

**Table 1:** Expression Profile and Differentiation Capacity of PrCSC s and hBM-MSCs

	FAP	CD90	CD105	CD73	aSMA	CD45	CD34	CD11b	CD19	HLADR	Adipocytes	Osteoblasts	Chondrocytes	Gleason Score
**hBM-MSC 1**	+	+	+	+	+	−	−	−	−	−	+	+	+	N/A
**hBM-MSC 2**	+	+	+	+	+	−	−	−	−	−	+	+	+	N/A
**PrCSC -1**	+	+	+	+	+	−	−	−	−	−	−	+	−	3+3
**PrCSC -2**	+	+	+	+	+	−	−	−	−	−	+	+	+	3+3
**PrCSC -3**	+	+	+	+	+	−	−	−	−	−	+	−	−	4+3
**PrCSC -4**	+	+	+	+	+	−	−	−	−	−	+	+	+	4+3
**PrCSC -5**	+	+	+	+	+	−	−	−	−	−	−	−	−	4+3
**PrCSC -6**	+	+	+	+	+	−	−	−	−	−	+	+	+	4+4

### Quantification of Mesenchymal Stem Cells in Human Prostate Cancer

**Figure 3:**
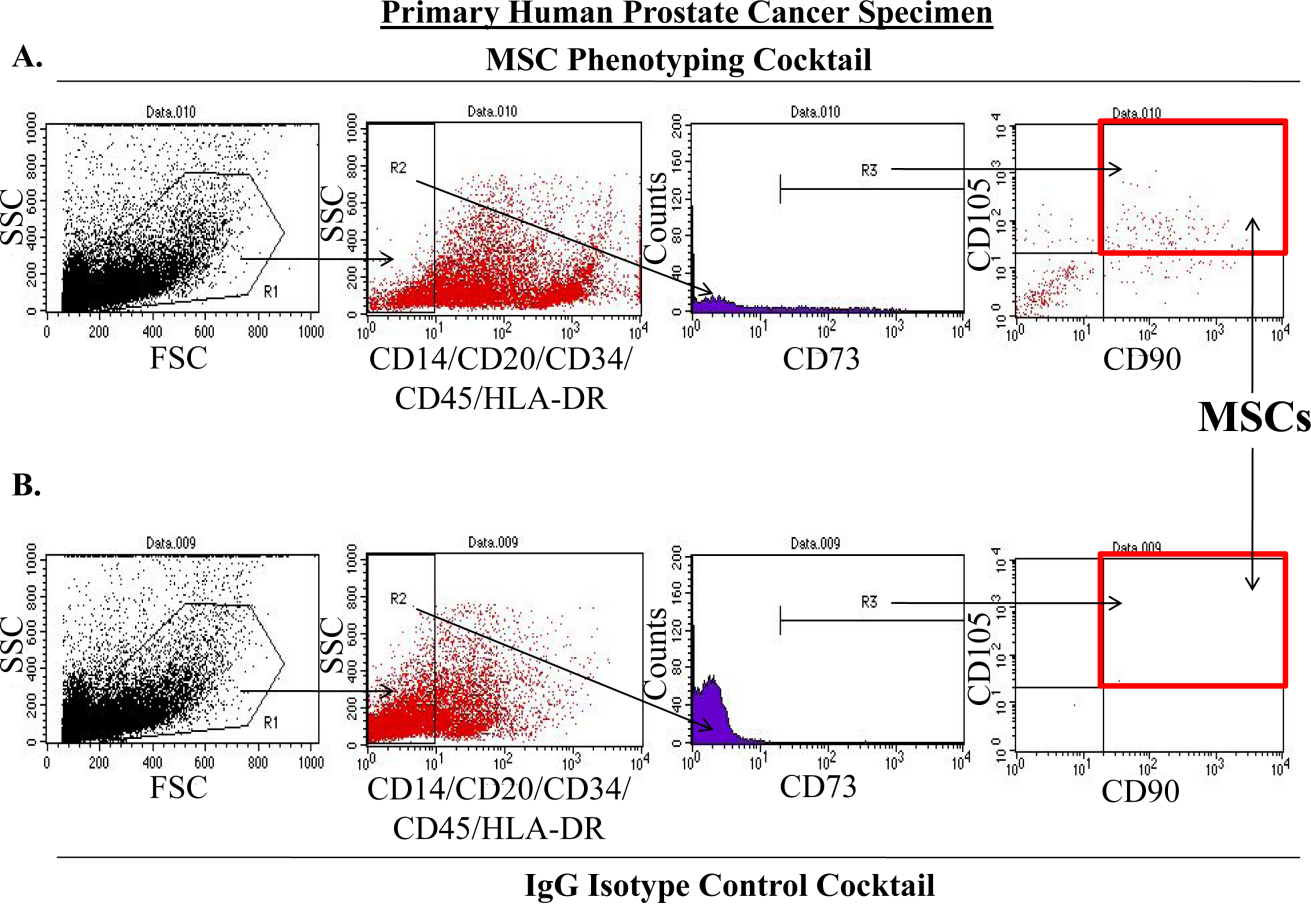
Method for Quantifying MSCs in Primary Human Prostatectomy Samples MSCs were quantified from primary human prostatectomy specimens using an optimized flow cytometry assay (A-B). Prostatectomy samples were digested into a single cell suspension using a combination of enzymatic and mechanical methods. At least 10,000 cells were initially gated (R1) on the basis forward and side scatter (FSC and SSC, respectively). From this initial population, lineage-negative cells (CD14^−^, CD20^−^, CD34^−^, CD45^−^, HLA-DR^−^) were selected (R2) and analyzed for expression of CD73 (R3). These lineage-negative, CD73-positive cells were further analyzed for the co-expression of CD90 and CD105. MSCs were defined as being lineage-negative and triple-positive for CD73, CD90, and CD105 (red box). Final quantification was performed by subtracting the number of events meeting these criteria in the IgG isotype control cocktail analysis (red box, B) from the events detected in the sample stained with the MSC phenotyping cocktail (red box, A). Importantly, all samples were analyzed within 3 hrs post-surgery.

To eliminate potential artifacts resulting from selection events associated with tissue culture, we optimized a flow cytometry-based assay to directly quantify the number of MSCs present in human prostate cancer samples directly from the patient prior to expansion in culture. Again, tissue cores of prostatectomy specimens were obtained immediately following surgery and digested into a single cell suspension using a combination of mechanical and enzymatic methods. Following labeling with either an MSC phenotyping cocktail (CD73, CD90, CD105, CD14, CD20, CD34, CD45, and HLA-DR) (Figure 3A) or an antibody isotype control cocktail (Figure 3B), these dissociated cells were analyzed by flow cytometry. MSCs within this population of cells were defined as being CD73, CD90, and CD105 triple-positive in the absence of CD14, CD20, CD34, CD45, and HLA-DR labeling (Figure 3). First, cells staining positive for the tested lineage markers were excluded from further analysis. Next, the CD73-positive cells within this lineage-negative population were selected. Finally, the number of MSCs present in the prostatectomy specimens were quantified by determining the number of CD73-positive, lineage-negative cells that were also double-positive for CD90 and CD105 (Figure 3). Of the 10 specimens analyzed in this study, MSCs represented between approximately 0.01 and 1.1% of the overall population of cells within the digested prostatectomy tissue (Table 2). As with the multi-lineage differentiation potential of PrCSCs derived from comparable prostatectomy specimens, there does not appear to be a relationship between the quantity of MSCs present in a particular sample and Gleason grade; however, the small number of samples characterized in this analysis preclude any conclusive judgments (Table 2). For comparison, CD31+ endothelial cells represented 1.89% of the cell population in the one prostatectomy specimen analyzed.

**Table 2:** Quantification of MSC s in Primary Human Prostatectomy Samples

Sample Gleason	Score	MSC s (%)
**PCa-1**	3+3	0.38
**PCa-2**	3+3	1.10
**PCa-3**	3+4	0.22
**PCa-4**	3+4	0.12
**PCa-5**	3+4	0.01
**PCa-6**	4+3	1.02
**PCa-7**	4+3	0.28
**PCa-8**	4+4	0.14
**PCa-9**	5+4	0.38
**PCa-10**	5+5	1.06

### Trafficking of Prostate Cancer-derived Stromal Cells & Mesenchymal Stem Cells to Prostate Cancer Xenografts

**Figure 4:**
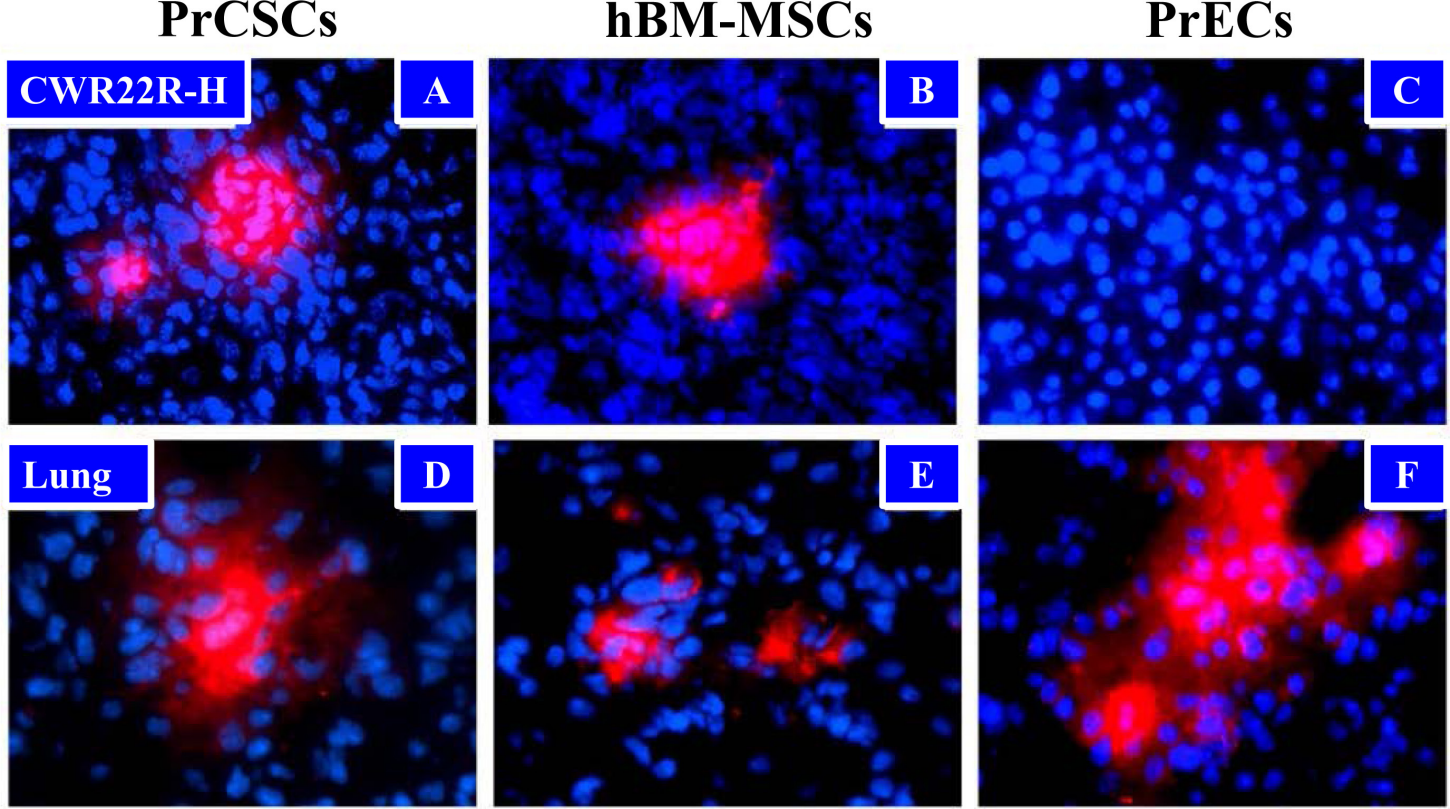
Tumor Trafficking of PrCSCs and hBM-MSCs to Human Cancer Xenografts in Mice PrCSCs (A) and hBM-MSCs (B), but not PrECs (C), traffic to prostate cancer xenografts in vivo following systemic infusion. Fluorescently-labeled (CM-DiI, red) PrCSCs, hBM-MSCs, and PrECs (1 × 10^6^) were infused intravenously (IV) into immunocompromised mice bearing subcutaneous CWR22RH xenografts (3/group). Four days post-infusion, lungs and tumors were harvested and analyzed by fluorescence microscopy for the presence of CM-DiI-labeled cells. In contrast to the xenografts, all three cell types were found entrapped in the lungs following infusion (D-F). Nuclei counterstained with DAPI (blue). At least three images analyzed per tissue per animal, representative images shown.

Additionally, hBM-MSCs are known to home to sites of cancer as a result of the inflammatory microenvironment present within these lesions. To determine whether PrCSCs also retained this ability, fluorescently-labeled cells (1×10^6^) were administered intravenously (IV) to animals bearing CWR22RH xenografts (3 animals/group). While all cell types tested (PrCSCs, hBM-MSCs, and PrECs) were found entrapped in the lungs at 4 days post-infusion (Figure 4A, B, and C), only the hBM-MSCs and PrCSCs were able to traffic to the prostate cancer xenograft (Figure 4D, E, and F).

## DISCUSSION

PrCSCs obtained directly from prostate cancer patients, prior to expansion in tissue culture, express CD90, CD73, and CD105 in the absence of CD14, CD20, CD34, CD45, and HLA-DR as demonstrated using an optimized flow cytometry assay (Figure 3, Table 2). Additionally, at least a subset of PrCSCs retains the ability to differentiate into osteoblasts, adipocytes, and chondrocytes (Figure 2). Therefore, PrCSCs fulfill all of the currently accepted criteria that are used to define MSCs. Importantly, both intra- and inter-patient heterogeneity is apparent in the population of cells isolated according to the current methods. This is demonstrated by the fact that not all PrCSC cultures isolated from different patients retained their multi-lineage differentiation potential (Table 1), and not all cells within a single culture were able to differentiate into all lineages under the appropriate induction conditions (Figure 2). Additionally, each core from each patient was heterogeneous with respect to the amount of cancer present, the number of cancer foci, and the degree of inflammation, all of which likely effect the number of MSCs quantified in any given specimen. Of the 10 prostatectomy specimens analyzed, the number of MSCs ranged from 0.01-1.1% of the overall cell population (Table 2). In comparison, CD31+ endothelial cells, which are known to play absolutely critical roles in tumorigenesis [29-30], represented 1-2% of the cells within sites of prostate cancer. Despite MSCs representing a relatively minor population of cells within the tumors analyzed, their numbers can reach approximately 50% of the endothelial cell content, and therefore, they may potentially play a significant role in prostate tumorigenesis. Interestingly, MSCs are often found in close association with blood vessels where there reside in a perivascular niche [9].

MSCs have previously been shown to influence carcinogenesis in a variety of ways, including promoting proliferation, angiogenesis, and metastasis, in addition to the generation of an immunosuppressive microenvironment [5, 7, 31]. Several studies have also shown MSCs to have anti-tumorigenic properties mediated through immunostimulatory properties and suppression of Akt- and Wnt-mediated survival signals [31-34]. Thus far, only a few studies have examined the role of MSCs in prostate carcinogenesis in vivo, which have predominantly demonstrated no effect on tumor growth [33, 35-41]. Unfortunately, these studies have primarily relied upon the PC3 cell line; therefore, experiments extending these observations into a broader range of models are necessary prior to making any conclusive judgments on MSCs role in prostate carcinogenesis. To further complicate the situation, MSCs have also been shown to give rise to so-called carcinoma-associated fibroblasts or CAFs [42-44], which have been the subject of many investigations into cancer and its relationship with the supporting stroma [45-50]. Our own data suggests that it is relatively easy to expand MSCs from primary human tissue samples under standard culture conditions, and these cells together with their progeny can quickly become a dominant population in the culture. Furthermore, these PrCSCs/MSCs express both aSMA and vimentin (Figure 1), the co-expression of which is commonly used to define reactive fibroblasts or CAFs [50]. This would suggest that many previous studies investigating the role of stromal cells derived from primary human tissue were actually studying MSCs depending on the passage used during the analysis and the frequency of MSCs in the starting population. While the mechanisms underlying the effects of MSCs in carcinogenesis are not fully understood, they are likely related to the complex relationship that exists between MSCs and the immune system [3, 5] coupled with the heterogeneity of tumor microenvironments and the cytokine profile present [18].

Chronic inflammation potentially resulting from a variety of stimuli, including dietary products, infectious agents, corpora amylacea-induced physical trauma, hormonal changes, and urine reflux, is frequently associated with prostate cancer precursor lesions [1-2]. The presence of *Mycoplasma hominis* has also been suggested as a cause of prostate inflammation [51]; however, these results may have been derived from tissue collection artifacts associated with transrectal biopsies [52]. Regardless of the cause, chronic inflammation has been suggested as an initiating event for prostate cancer [1-2]. Additionally, prostate cancers typically express high levels of pro-inflammatory chemokines, including CXCL12 (SDF-1), CCL5 (RANTES), and CCL2 (MCP-1) [20-22]. The expression of these cytokines has been shown to mobilize systemic reservoirs of inflammatory and immunomodulatory cells, including BM-MSCs, which are recruited to prostate cancer lesions [17-18]. MSCs express an extensive array of cytokine receptors, which have been shown to mediate their trafficking to sites of inflammation and cancer [17]. Furthermore, MSCs also secrete a large number of immunomodulatory, growth, and signaling molecules, including TGF-B, GM-CSF, RANTES, CCL2, VEGF, HGF, IL-6, and IL-10 [3-4, 19, 53], which may help to initiate a self-reinforcing loop that may lead to chronic inflammation under pathological conditions and contribute to carcinogenesis. Given the regenerative, immunomodulatory and immunotrafficking properties of MSCs, it is not surprising to find these cells in the prostate during tissue regrowth [54], carcinogenesis (Figure 3, Table 2), and inflammation-associated pathologies, such as BPH [23]. Placencio et al. have previously demonstrated that bone marrow-derived MSCs contributed to prostate regrowth following testosterone supplementation in mice post-castration [54]. Previous work by Lin et al. has also demonstrated that stromal cells consistent with an MSC phenotype from older donors can be isolated from BPH tissue [23]. The authors concluded that these stromal cells did not represent MSCs due to their inability to generate neurons, a property that has been shown to decrease with the age of the donor [13, 24], and therefore, is likely explained by the prevalence of BPH in older men from which the tissue analyzed was obtained. An earlier study comparing CD90hi vs. CD90lo primary stromal cells isolated from prostate cancer patients also concluded that these cells did not represent MSCs [55]. However, it should be noted that the differentiation potential of these cells was not assayed. Furthermore, CD90hi cells were compared to CD90lo cells rather than CD90neg cells, both of which may represent MSCs at different stages of differentiation and would explain the observed similarities in their expression profiles. The data presented herein clearly demonstrates that there is a rare population of CD90-positive MSCs present in tissue isolated from primary prostate cancer patients (PrCSCs) (Figures 2-3, Table 2).

As described above, there is extensive literature demonstrating that BM-MSCs can home to sites of prostate cancer based upon the inflammatory microenvironment present within these lesions [3, 17-19]. Due to the lack of HLA-DR expression and immunologic co-stimulatory molecules, these cells are non-immunogenic even in an allogeneic setting [3-4, 16]. This suggests that MSCs can be used to systemically deliver therapeutic or imaging agents to both primary and metastatic prostate cancer deposits throughout the body. Additionally, our data suggests that PrCSCs retain this tumor trafficking ability as well (Figure 4), which raises the possibility of using autologous cells derived from a patient's own prostatectomy specimen to target systemic disease; however, ethical concerns related to infusing patients with autologous cancer-associated stromal cells would be of significant concern with this latter approach. Much previous work has attempted to exploit the tumor-trafficking properties of MSCs derived from a variety of non-malignant sources to deliver cytotoxic agents to various solid tumor types with mixed results [56-60]. Importantly, these studies failed to take into account that MSCs traffic to other sites throughout the body, including the lungs, bone marrow, and spleen, in addition to the tumor; therefore, dose-limiting toxicities can be manifested from the delivery of these compounds to peripheral non-target tissues. To circumvent this problem, a prodrug approach exploiting tumor- or tissue-selective activation of a therapeutic compound in which the MSCs were used as a vector to enhance drug accumulation within the tumor would potentially be of greater therapeutic benefit. Additionally, studies by Sarkar et al. have demonstrated that cell engineering approaches can be used to enhance the homing and engraftment efficiency of MSCs in target tissues by mimicking mechanisms of leukocyte extravasation [61].

In summary, primary human prostate cancer harbors a population of cells consistent with MSCs. Stromal cells derived from human prostatectomy specimens (PrCSCs) share an expression profile with MSCs derived from the bone marrow (BM-MSCs) for all cell surface markers analyzed. Like BM-MSCs, these PrCSCs have the ability to differentiate into adipocytes, osteoblasts, and chondrocytes; thereby, demonstrating their multi-lineage differentiation potential. Both BM-MSCs and PrCSCs are able to traffic to prostate cancer xenografts in vivo, likely as a result of the pro-inflammatory cytokine and chemokine milieu present. Therefore, MSCs represent a potential drug delivery vector for future therapeutic approaches targeting both local and metastatic prostate cancer.

## METHODS

### Reagents

Rat anti-human CD11b-APC (clone M1/70.15.11.5), mouse anti-human CD19-PE (clone LT19), mouse anti-human CD34-PE (clone AC136), mouse anti-human CD45-APC (CLONE 5B1), mouse anti-human CD326(EpCAM)-FITC (clone HEA-125), mouse anti-human CD326(EpCAM)-PE (clone HEA-125), mouse anti-human CD326(EpCAM)-APC (clone HEA-125), and mouse anti-human HLA-DR-PerCP (clone AC122) antibodies were purchased from Miltenyi Biotec, Inc. (Bergisch Gladbach, Germany). Mouse anti-human HLA-DR-APC (clone LN3), mouse anti-human CD73-APC (clone AD2), mouse anti-human CD105-PE (clone SN6), mouse anti-human CD326(EpCAM)-biotin (clone 1B7) and mouse anti-human FAP (clone F11-24) were purchased from eBioscience (San Diego, CA). Mouse anti-human CD90-FITC (clone F15-42-1) was purchased from Millipore (Billerica, MA). Mouse anti-human aSMA-FITC (clone 1A4) was purchased from Abcam (Cambridge, MA). Mouse anti-human CK5 (clone XM26) was purchased from Vector Laboratories (Burlingame, CA). Mouse anti-human CK8 (clone LP3K) was purchased from Santa Cruz (Santa Cruz, CA). Mouse anti-human vimentin (clone LN-6) was purchased from Sigma-Aldrich (St. Louis, MO). Goat anti-mouse Alexa Fluor 488, Roswell Park Memorial Institute (RPMI)-1640 medium, keratinocyte-serum free medium (K-SFM), Hank's Balanced Salt Solution (HBSS), L-glutamine, and penicillin-streptomycin were purchased from Life Technologies-Invitrogen (Carlsbad, CA). Fetal bovine serum (FBS) was purchased from Gemini Bioproducts (West Sacramento, CA).

### Primary Cell Isolation and Tissue Culture

hBM-MSCs were obtained from Lonza (Walkerville, MD). Primary prostate epithelial and stromal cells from patient radical prostatectomy specimens were isolated at our institution in accordance with an Institutional Review Board approved protocol according to previously published protocols [25-28, 62] for the cell cultures used in the differentiation assays, immunofluorescence staining, and cell surface expression studies. hBM-MSCs and PrCSCs were cultured in RPMI-1640 medium supplemented with 10% FBS, 1% L-glutamine, and 1% penicillin-streptomycin in a 5% CO_2_, 95% air humidified incubator at 37°C. PrECs were grown in K-SFM with defined growth factors [25-28] in the same 5% CO_2_, 95% air humidified incubator at 37°C.

### Immunofluorescence

Immunofluorescent staining for aSMA, Vim, CK5, and CK8 were performed using the antibodies listed above according to previously published protocols [27-28]. Nuclei are counterstained with DAPI using ProLong Gold anti-fade with DAPI (Invitrogen). Images were captured using a Nikon (Melville, NY) Eclipse Ti Fluorescent scope equipped with a Nikon DS-Qi1Mc camera NIS-Elements AR3.0 imaging software.

### Multilineage Differentiation

To assay adipogenic differentiation, 2 × 10^5^ cells were plated in a 6-well plate and allowed to reach 100% confluence (3 replicates/cell type) in an incubator with 5% CO_2_ at 37°C. The media was then changed to hMSC adipogenic induction medium (Lonza) supplemented with h-insulin (recombinant), L-glutamine, MCGS, dexamethasone, indomethacin, IBMX (3-isobuty-l-methyl-xanthine), GA-1000. According to the manufacturer's instructions, media was changed every three days alternating between induction and maintenance medium for three complete cycles. After the final cycle, cells remained in the maintenance medium for an additional 7 days prior to evaluation of adipogenic differentiation. Negative control cells were grown in maintenance media only. Adipogenic differentiation was assayed using the lipid stain Oil Red O (Sigma) to identify lipid vacuoles in differentiated cells.

To assay osteogenic differentiation, 3 × 10^4^ cells were plated in a 6-well plate and allowed to adhere overnight at 37°C in an incubator with 5% CO_2_ (3 replicates/cell type). According to the manufacturer's instructions, the media was then changed to Osteogenic Induction media (Lonza) supplemented with dexamethasone, L-glutamine, ascorbate, MCGS, b-glycerophosphate. Media was changed every 3-4 days for 21 days. Negative control cells were cultured in RPMI-1640 supplemented with 10% FBS, L-glutamine, penicillin-streptomycin. After 21 days, osteogenic differentiation was assayed by staining for calcium deposits with Alizarin Red S (Sigma).

To assay chondrogenic differentiation, 2.5 × 10^5^ cells were centrifuged at 150 × g for 5 min at room temperature and resuspended in 0.5 mL chondrogenic induction medium (Lonza) supplemented with dexamethasone, ascorbate, ITS, GA-1000, sodium pyruvate, proline, L-glutamine, and TGF-B3 in a 15 mL polypropylene conical tube according to the manufacturer's instructions (3 replicates/cell type). The caps were loosened a half-turn and placed at 37°C in an incubator with 5% CO_2_. The media was changed every 3 days for 21 days while being careful to avoid aspirating the pellet. After 21 days, cell pellets were fixed in formalin and paraffin-embedded for histological processing. Negative controls were cultured in the absence of TGF-B3. Chondrogenic differentiation was assayed by staining for glycosaminoglycans with Safranin-O (Sigma).

### Analysis of Cell Surface Markers and MSC Quantification by Flow Cytometry

To analyze cell surface marker expression, prostatectomy cores were dissociated into a single cell suspension as described previously [25-28, 62]. Flow cytometry analyses were also performed as described previously [26-27]. Briefly, all antibody incubations, washes, and flow cytometric analyses were performed in MACS cell sorting buffer (Miltenyi). Antibody labeling was performed at 4°C for 20 min with a 1:10 dilution of the antibody in a volume of 100 μl per 1×10^6^ cells. The cells were washed in 1 mL cold cell sorting buffer, resuspended in 1.0 mL cell sorting buffer and passed through a 0.2 m filter into a flow analysis tube (BD Biosciences, Franklin Lakes, NJ). Analysis was performed on a BD FACSCalibur flow cytometer.

To obtain cell suspensions for quantification of MSCs by flow cytometry prior to expansion in tissue culture, the following protocol was optimized. Twenty-five 18-gauge biopsy needle cores (C. R. Bard, Inc., Tempe, AZ) were obtained and washed in HBSS. Five randomly selected cores were fixed, paraffin-embedded, and sectioned for H&E staining and pathological confirmation. The remaining cores were digested using a human tumor dissociation kit (Miltenyi) and a gentleMACS dissociator (Miltenyi) according to the manufacturer's instructions. The dissociated cell suspension was then passed through a 70 um pre-separation filter (Miltenyi). The sample was centrifuged at 250 × g for 5 min and resuspended in RBC lysis buffer (Miltenyi) for 10 min at room temperature. The RBC-negative cell suspension was centrifuged at 250 × g for 5 min and resuspended in MACS cell sorting buffer (Miltenyi) to determine cell number and viability by trypan exclusion using a Cellometer Auto T4 (Nexcelcom Bioscience, Lawrence, MA) prior to downstream flow cytometry applications.

All antibody incubations, washes, and flow cytometric analyses were performed in MACS cell sorting buffer (Miltenyi). Antibody labeling was performed at 4°C for 10 min with a 1:10 dilution of with a MSC Phenotyping Cocktail (anti-CD14-PerCP, anti-CD20-PerCP, anti-CD34- anti-PerCP, anti-CD45-PerCP, anti-CD73-APC, anti-CD90-FITC, and anti-CD105-PE) or Isotype Control Cocktail (Mouse IgG1-FITC, Mouse IgG1-PE, Mouse IgG1-APC, Mouse IgG1-PerCP, and Mouse IgG2a-PerCP) provided in the human MSC phenotyping kit (Miltenyi) in a volume of 100 ul per 1×10^6^ cells according to the manufacturer's instructions. Additionally, anti-HLA-DR-PerCP (Miltenyi) was added to the MSC Phenotyping Cocktail. The cells were washed in 1 mL cold cell sorting buffer, resuspended in 0.5 mL cell sorting buffer and passed through a 0.2 um filter into a flow analysis tube. Analysis was performed on a BD FACSCalibur flow cytometer. All compensation controls were performed using anti-EpCAM antibodies directly conjugated to FITC, PE, APC, or Biotin followed by anti-Biotin-PerCP on aliquots of the same cell suspension to ensure proper gating and instrument settings prior to sample analysis. For sample analysis, cell suspensions labeled with either the Isotype Control or MSC Phenotyping Cocktails were gated (R1) on the basis of forward and side scatter (FSC & SSC, respectively) (Figure 3). Cells gated in R1 were then selected based on being lineage-negative (R2), i.e., negative for CD14, CD20, CD34, CD45, and HLA-DR expression. Next, CD73-positive cells (R3) within these lineage-negative cells were further analyzed for co-expression of CD90 and CD105. MSCs were defined as cells that were triple-positive for CD90, CD73, and CD105 in the absence of the tested lineage markers and quantified by subtracting the number of events, if any, that met these criteria in the isotype control sample. This corrected number was used as the numerator to determine the percentage of MSCs present in the sample. At least 10,000 events were collected in R1, which defined the number of total cellular events and was used as the denominator in the above calculation. Importantly, all samples were processed and analyzed within 3 hrs post-surgery.

### Cell Trafficking to Prostate Cancer Xenografts in vivo

Animal studies were performed according to protocols approved by and performed in accordance with the guidelines of the Animal Care and Use Committee of the Johns Hopkins University School of Medicine. For CWR22RH xenografts, 50 mg of minced tumor tissue that had passed through a sterile tissue strainer and washed with HBSS was implanted subcutaneously in 100 ul of 80% Matrigel (BD Biosciences, Sparks, MD) in the flanks of NOG-SCID mice.

To assay tumor trafficking, human PrCSCs, hBM-MSCs (Lonza), or PrECs were fluorescently-labeled ex vivo with CM-DiI (Invitrogen) and washed according to the manufacturer's instructions. Subsequently, 1×10^6^ cells were injected intravenously into NOG-SCID mice bearing subcutaneous CWR22RH tumors (3 mice/group). Animals were euthanized by CO_2_ asphyxiation at 4 days post-infusion. The lungs and tumors were harvested from each mouse, flash frozen in VWR Clear Frozen Section Compound (Radnor, PA), and 4 μm sections were cut on a Shandon Cryotome E (Thermo Scientific, Waltham, MA). Nuclei are counterstained with DAPI using ProLong Gold anti-fade with DAPI (Invitrogen). Images were captured using a Nikon (Melville, NY) Eclipse Ti Fluorescent scope equipped with a Nikon DS-Qi1Mc camera NIS-Elements AR3.0 imaging software.
